# Chronic Anxiety- and Depression-Like Behaviors Are Associated With Glial-Driven Pathology Following Repeated Blast Induced Neurotrauma

**DOI:** 10.3389/fnbeh.2021.787475

**Published:** 2021-12-10

**Authors:** Michelle R. Dickerson, Susan F. Murphy, Michael J. Urban, Zakar White, Pamela J. VandeVord

**Affiliations:** ^1^Department of Biomedical Engineering and Mechanics, Virginia Tech, Blacksburg, VA, United States; ^2^Salem VA Medical Center, Salem, VA, United States

**Keywords:** blast, TBI, neuropsychiatric, hippocampus, motor cortex, astrocytes, dendrites

## Abstract

Long-term neuropsychiatric impairments have become a growing concern following blast-related traumatic brain injury (bTBI) in active military personnel and Veterans. Neuropsychiatric impairments such as anxiety and depression are common comorbidities that Veterans report months, even years following injury. To understand these chronic behavioral outcomes following blast injury, there is a need to study the link between anxiety, depression, and neuropathology. The hippocampus and motor cortex (MC) have been regions of interest when studying cognitive deficits following blast exposure, but clinical studies of mood disorders such as major depressive disorder (MDD) report that these two regions also play a role in the manifestation of anxiety and depression. With anxiety and depression being common long-term outcomes following bTBI, it is imperative to study how chronic pathological changes within the hippocampus and/or MC due to blast contribute to the development of these psychiatric impairments. In this study, we exposed male rats to a repeated blast overpressure (~17 psi) and evaluated the chronic behavioral and pathological effects on the hippocampus and MC. Results demonstrated that the repeated blast exposure led to depression-like behaviors 36 weeks following injury, and anxiety-like behaviors 2-, and 52-weeks following injury. These behaviors were also correlated with astrocyte pathology (glial-fibrillary acid protein, GFAP) and dendritic alterations (Microtubule-Associated Proteins, MAP2) within the hippocampus and MC regions at 52 weeks. Overall, these findings support the premise that chronic glial pathological changes within the brain contribute to neuropsychiatric impairments following blast exposure.

## Introduction

Traumatic brain injury (TBI) is a leading cause of death and disability in trauma patients and is a significant clinical challenge within the active duty and Veteran populations. Blast exposure has been credited for the substantial number of trauma injuries and blast-induced TBI (bTBI) has been reported as the most prevalent injury in recent military conflicts (Report to Congress: Traumatic Brain Injury in the United States | Concussion | Traumatic Brain Injury | CDC Injury Center, 2019). Between 2000 and 2019, 413,858 United States Soldiers and Veterans sustained a TBI with 82.8% of those TBIs being considered mild (Acosta et al., 2013; Traumatic Brain Injury, 2014). Additionally, mild bTBI has been shown to have an “invisible nature” as there are very few, if any, physical presenting signs. Because of this, Soldiers may return to the field, making them susceptible to sustaining further injuries (Carr et al., 2015; Kamimori et al., 2018).

Mild bTBI is associated with acute deficits, with the majority of patients showing a full recovery with time. However, a subset of mild bTBI patients was shown to have long-lasting and debilitating effects (Theeler et al., 2012; Agoston, 2017). Chronic pain and headache conditions are among those most frequently reported by Veterans and are rationalized as predecessors for cognitive and behavioral disorders of bTBI (Zaloshnja et al., 2008; Gavett et al., 2010). The outcomes of cognitive and behavioral deficits include memory loss, fear, anxiety, depression, and/or lack of problem-solving skills that can lead to analgesia/opioid abuse and suicide (Baalman et al., 2013; Bjork et al., 2016; Fakhoury et al., 2020). While attention to these comorbidities has been a focus of research to improve Veteran healthcare, reports on Veterans with chronic behavioral, psychiatric symptoms are increasing (Alexis et al., 2008; Alway et al., 2012, 2016; Higgins et al., 2014; Badea et al., 2018).

An estimated 3.17 million Americans are diagnosed with TBI-induced neuropsychiatric impairments [many of which are related to post-traumatic stress disorder (PTSD; Dieter and Engel, 2019; Denby et al., 2020)], presenting a growing challenge for the advancement of healthcare for our civilian and military populations (Bjork et al., 2016; Lim et al., 2017). Depression, being the least studied neuropsychiatric impairment in Veterans, is one of the most commonly reported (Miles et al., 2017), and a significant comorbidity (76%) with anxiety has also been reported (Jorge et al., 2004). Veterans who develop a depressive disorder following bTBI are known to have more functional impairments, leading to a complicated recovery as compared to those with TBI histories and no reports of psychiatric disorders (Hesdorffer et al., 2009). Further, Veterans with depressive disorders are reported to develop social anxiety, the fear of interacting or doing things with or in front of other people (Kashdan et al., 2006). This lack of sociability can lead to avoidance behavior, where individuals avoid or act in a way that prevents being humiliated or rejected. Depression can also lead to consequences associated with poor physical health, diminished functional capacity, and impaired executive control (Hall et al., 2014). These outcomes diminish the quality of life of Soldiers and Veterans diagnosed with depression. A retrospective analysis of clinical studies examining depression at least 6 months post-injury in adult subjects with mild TBI of any etiology, including civilians and military, found that mild TBI was associated with a 3.29-fold increased risk of depression. They further found that a similar risk of depression was sustained across different ages at injury and that depression was present across all etiologies studied (Hellewell et al., 2020). Thus, concluding that those who experience a mild TBI are three times more likely to experience depression compared to those without a history of mild TBI, and this risk persists years beyond the TBI event.

Repetitive concussions are associated with long-term neurodegenerative changes which result in behavioral impairments such as depression (Bugay et al., 2020). Concussive blast forces have unique biomechanical features compared to other closed-head injuries, thus studying the mechanistic changes that contribute to neurodegeneration may give insight into the common behavioral outcomes seen in those diagnosed with bTBI. Like traditional neuropathology resulting from impact, astrocyte reactivity, neuronal dysfunction, and blood-brain barrier (BBB) disruption have been described (Sajja et al., 2012; Karve et al., 2016; Abrahamson and Ikonomovic, 2020). The hippocampus is an important part of the limbic system and has been demonstrated to be susceptible to blast injury (Cho et al., 2013; Sajja et al., 2014). Injury to the hippocampal neurons has also been linked to contributing to depressive behaviors but has not been studied extensively following blast injury. Molecular and cellular studies have examined the intracellular signaling pathways involved in the regulation of synaptic function by stress. Notably in the hippocampus, decreased neuroplasticity and synapse function in the dentate gyrus and CA sub-regions have been associated with causing depression-like behaviors (Law et al., 2016; Hao et al., 2020). Dendrites, for example, receive messages for the cell, allowing neurons to communicate with other neurons. Blast injury may contribute to dendritic dysfunction within the hippocampus resulting in neuropsychiatric impairment. Studies have also identified that pathology within the motor cortex (MC) may also play a role in the manifestation of depression. While the MC is commonly associated with cognition, it is hypothesized that damage to the circuitry in the MC implicated with cognition may result in depressive-like symptoms (Pennisi et al., 2016). A review by Northoff et al. (2021) reported that cognitive deficits due to neuronal damage in the MC played a prominent role in the progression of major depressive disorder (MDD). With these impairments being prevalent in neuropsychiatric disorders, and with Veterans reporting depression-like symptoms following blast injury, a fundamental understanding of the relationship between the MC and chronic depression is vital to advancing the field.

While damage to neural circuitry in the hippocampus and/or MC has been shown to have an impact on depression (Rao et al., 2019), a focus on how the glial cells contribute to the morbidities is lacking. Astrocytes associated with injured tissue are often termed to be reactive, or astrogliotic. While the timing of altered expression may vary and possibly depict stages of injury recovery, intermediate filaments such as glial-fibrillary acid protein (GFAP) and vimentin are known to increase in response to injury (Ekmark Lewén et al., 2010; Schwerin et al., 2021). In what is designated the tripartite synapse, astrocytes contribute to bidirectional communication between neurons. Because of the crucial role that astrocytes play in neuronal integrity, signaling damage of neurons will often cause astrocytes to become reactive (Farhy-Tselnicker and Allen, 2018; Zhou et al., 2020). Recent studies have investigated chronic behavioral outcomes such as anxiety-like behaviors following bTBI, with studies ranging from the 6 to 13 months following injury (Arun et al., 2020; Blaze et al., 2020). They found significant neuromotor and anxiety-like behaviors associated with amygdala pathology. To our knowledge, studies examining the pathological changes associated in the hippocampus and/or MC following blast injury and how they contribute to depression-like behaviors are limited. With anxiety also being a chronic behavioral outcome, understanding whether the hippocampus and/or MC play a role in anxiety-like behaviors is also of interest. As these comorbidities can be symptoms of PTSD, these behavioral deficits can be misdiagnosed and treated as PTSD rather than as a result due to blast exposure (Bryant, 2011). Because the injury mechanisms that result in chronic anxiety and depression following repeated bTBI are uncertain, preclinical investigations are needed to bridge the significant knowledge gap. This study aimed to characterize the chronic neuropathological changes within the hippocampus and MC while measuring levels of anxiety- and depressive-like behaviors up to 52 weeks using a rodent model of repeated blast exposure. It was hypothesized that chronic pathology in the hippocampus and/or MC would contribute to the subsequent behavioral phenotypes.

## Materials and Methods

### Animal Procedures and Blast Exposure

All experimental protocols described within this study were approved by the Virginia Tech Institutional Animal Care and Use Committee. Additionally, all experiments were performed in accordance with relevant guidelines and regulations. Prior to all experiments, male 10-week-old Sprague Dawley rats (Envigo, Dublin, VA) weighing 275.6 ± 10.5 g were acclimated for several days (12 h light/dark cycle) with food and water provided *ad libitum*.

Blast injuries were performed as described in Dickerson et al. (2020). Briefly, the blast wave was generated using a custom Advanced Blast Simulator (ABS; 200 cm × 30.48 cm × 30.48 cm) located at the Center for Injury Biomechanics at Virginia Tech University. The ABS consisted of three distinct sections to create, develop, and dissipate the blast wave. The blast wave developed following a helium-driven rupture of calibrated acetate membranes. The passive end-wave eliminator was located downstream of the test location to facilitate the dissipation of the blast wave through a series of baffles. As a result, the test location was exposed to a single peak overpressure representing a free-field blast exposure. Pressure measurements were collected at 250 kHz using a Dash 8HF data acquisition system (Astro-Med, Inc., West Warwick, RI, USA). Analysis of pressure profiles was conducted using a custom MATLAB script to calculate the impulse and duration of the positive and negative phases and rise time. Peak overpressure was determined using the Rankine–Hugoniot relations and observed wave speed at the animal test location within the ABS.

In preparation for blast exposure, animals were anesthetized with 5% isoflurane and placed inside the ABS where each animal was supported in the prone position facing the oncoming shock front using a mesh sling. The sling was designed to minimize the hindrance of flow and to isolate blast injury by limiting motion, restricting the animals from impacting the walls of the simulator. Animals were exposed to three static overpressure insults (16.40 ± 1.54) separated by 1 h each (3 × 1 h; *n* = 11). A sham group (*n* = 10) received all the same procedures except for the blast exposures. All animals were observed through the recovery stages of injury and anesthesia.

### Weight Monitoring and Food Restrictions

For this long-term study, animals were monitored over 52 weeks making it important to introduce weight monitoring and food restrictions to ensure the animals maintained a healthy weight. The weight of the animals was monitored every 4 weeks up to 24 weeks post-blast. At week 24 post-blast, food restrictions were started, and animals were weighed weekly. Healthy adult male Sprague-Dawley rats typically eat up to 25 g of rodent chow a day, therefore 25 g/per rat of chow was weighed and placed in each cage daily. Any uneaten food was removed and weighed. The amount of food eaten, and weights were recorded. Additionally, a variety of environmental enrichment items were made available throughout the study.

### Elevated Plus Maze (EPM)

EPM was used as a measure of anxiety, which is based on the conflicting drive of rats to explore novel environments, avoiding open areas and aversive threats. The apparatus consists of a “+” shaped maze, in which two open arms (50 × 10 × 50 cm) are intersected by two enclosed arms (50 × 10 × 30 cm enclosures) at a center square (10 × 10 × 50 cm) region in an isolated room under low light (~6 lux). An overhead low-light camera recorded behavior at 30 frames per second. Animals were placed in the center of the maze facing the same open arm (away from the investigator) and 5-min recordings were initiated as the investigator exited the room. Video files were captured and scored *via* EthoVision XT^TM^ (Noldus Information Technology, Leesburg, VA), tracking software using three-point tracking.

### Three-Chamber Sociability Test

The three-chamber sociability test was performed 36 weeks following repeated blast exposure. This test is carried out in two sequential, 10-min trials. First, the rat is given a 10-min habituation period with the doors open and both cages empty. For the sociability test, the test rat is immediately placed in the center chamber (20 × 40 cm) with the doors in place and an unfamiliar rat (Stranger I) is placed in one of the side chambers (30 × 40 cm) enclosed in a cage with clear acrylic bars that allowed nose contact between the bars (sniff zone: 16 × 16 cm). The opposite chamber contained an identical, empty cage. The test rat is allowed to explore the entire arena for 10 min. For analysis, a discrimination index was calculated for each trial (time spent in the stranger’s chamber divided by the total time spent in the empty and stranger’s chamber). A ratio of 0.5 indicated equal exploration in each chamber, with a ratio less than 0.5 showing sociability deficits, and greater than 0.5 indicating sociability.

### Immunohistochemistry (IHC)

Fifty-two weeks following repeated blast exposure, animals were anesthetized with 5% isoflurane and euthanized by transcardial perfusion: first with 0.9% saline then 4% paraformaldehyde. Brains were extracted and post-fixed for 24 h to ensure proper fixation, followed by being rinsed in phosphate buffer saline (PBS) and dehydrated in a 30% sucrose solution. Fixed brains were then embedded and frozen in optimal cutting temperature medium (OCT; Sakura Finetek Inc., Torrance, CA, USA) at 80°C. Coronal sections (40 μm) were prepared with a cryostat microtome (Thermo Scientific Inc., Waltham, MA) and stored in PBS with 0.05% sodium azide at 4°C prior to staining procedures. IHC was performed on selected sections containing specific regions of interest: MC and the hippocampus which was delineated into the CA1, CA2, CA3, and DG regions. Sections were stained with the following antibodies: Glial Fibrillary Acidic Protein (GFAP), Microtubule-Associated Proteins (MAP2), NeuN, and Vimentin (Table 1). Tissue sections were first rinsed with PBS containing 0.03% Triton-X (PBX) and incubated in either 2% bovine serum albumin (GFAP), 10% normal goat serum (MAP2, NeuN), or 5% normal donkey serum (Vimentin) for 1 h. Sections were then incubated in primary antibody diluted in blocking buffer for 16–18 h at 4°C. Samples were washed with PBX and then incubated with secondary antibodies: Alexa Flour 488 anti-mouse IgG antibody (GFAP, NeuN) or Alexa Flour 546 anti-rabbit IgG antibody (MAP2, Vimentin). Following incubation, samples were again washed with PBX and then mounted on slides, air-dried, and cover slipped with Prolong Antifade Gold Mountant (Invitrogen, Carlsbad CA) reagent with 6-diamidino-2-phenylindole (DAPI; Invitrogen, Carlsbad, CA, USA). Sections were examined and imaged by an investigator blinded to the treatment group using a Zeiss fluorescence microscope at 20× magnification (Zeiss, Jena, Germany).

**Table 1:** Primary antibodies used for histological analyses.

Antibody	Catalog number	Vendor
GFAP (1:500)	13-300	Invitrogen (Carlsbad, CA)
NeuN (1:500)	MAB377	EMD Millipore (Burlington, MA)
MAP2 (1:1,000)	NB300-213	Novus Biologicals (Littleton, CO)
Vimentin (1:500)	ab92547	Abcam (Cambridge, MA)

Area fraction, count per area, fluorescence intensity, and mean area per cell were the four specific parameters quantified for image analysis using ImageJ software (National Institutes of Health, Bethesda, MD). Area fraction was used to quantify the percentage of positive signals within the region of interest, while count per area represented the total number of positive cells divided by the area of the region of interest. Fluorescence intensity measured the positive signal of an image using the gray pixel intensity, hence, analyzing the level of expression of the protein of interest. Lastly, the mean area per cell provided detail on the average cell soma size normalized to the area. Both the count per area and area per cell were completed by using the particle size exclusion function “analyze particles” on ImageJ. This function took a pixel area size threshold of 0.004 (NeuN), 0.04 (GFAP), or 0.09 (Vimentin) to exclude small pixel noise and extract objects of interest. Mean brain region values were derived from four images for each animal per stain.

### Statistical Analysis

All statistical analyses were performed in GraphPad Prism version 9 software (GraphPad Software, La Jolla, CA). A one-way ANOVA was used to assess the difference between time spent in the three chambers for both the sham and blast groups. For all histological data and additional behavior assay data, a student’s *T*-test was used to compare blast and sham groups. Outliers were identified by calculating the studentized residuals, excluding data points above −2 or 2. The Shapiro-Wilk test and Levene’s test were used to verify assumptions of normality and homoscedasticity, respectively. In the instances that data failed these assumptions, either Welch’s correction t-test or Mann-Whitney’s non-parametric test were performed. Data were considered statistically significant with *p* < 0.05 and trending at *p* < 0.1. All histology data were normalized to respective shams. All data are presented as the mean ± standard error of the mean (SEM). Finally, the potential relationship between histology and behavior was determined using correlation analysis (Pearson’s R test).

## Results

### Blast Event and Physiological Outcomes

**Figure 1:**
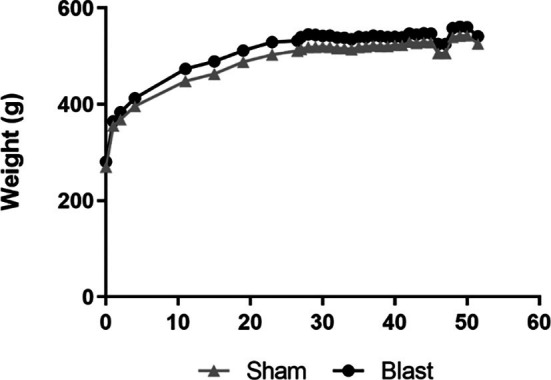
Change in weight over 52 weeks in sham and blast groups. At week 24, food restrictions began. Data is represented as Mean ± SEM.

Blast animals were exposed to three blast events 1 h apart. Blast wave characteristics are described in Table 2. Following each blast exposure, no obvious external signs of injury were discernable. Because of the long-term study, weight maintenance was required. Once animal weights reached 500 g, the amount of food per day was reduced to 17.5–20 g to maintain their weight and minimize weight gain (Figure 1). At 23 weeks the mean weight was 505.7 g ± 35.28 (SEM). At 52 weeks, the mean weight was 534.9 g ± 28.64. Hence, a healthy weight range was maintained and no difference was seen between treatment groups.

**Table 2:** Blast wave characteristics.

Injury	Peak pressure (psi)	Duration (ms)	Impulse (psi*ms)	Rise time (ms)
3 × 1 h Blast	16.40 ± 1.54	2.19 ± 0.15	13.03 ± 1.20	0.03 ± 0.033

*Ten-week-old Sprague Dawley rats weighing 275.6 ± 10.5 g were anesthetized and exposed to three blast insults separated by 1 h each. Sham animals underwent all procedures with the exception of the blast insult. The average peak pressure resulted in a blast wave magnitude of approximately 17 psi. Results are represented as Mean ± SEM*.

### Anxiety-Like Behaviors Are Observed 2- and 52-Weeks Following Blast Injury

**Figure 2:**
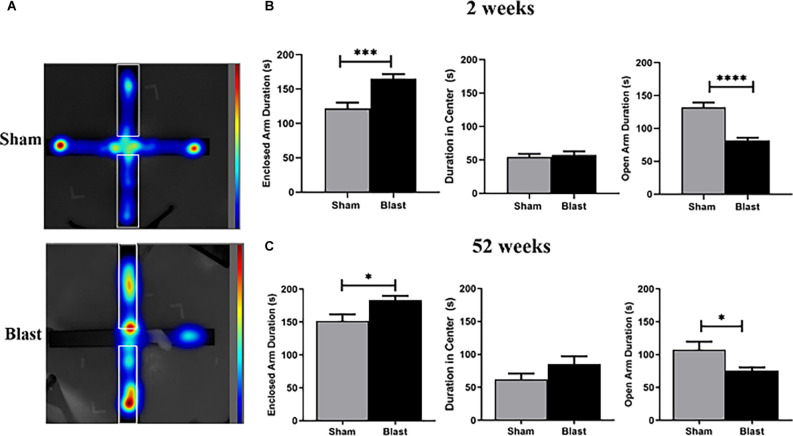
Animals exhibit anxiolytic behavior 2- and 52-weeks following injury. **(A)** Representative heat map images show that blast animals spent more time in the enclosed arm (outlined area) than their sham counterparts. **(B)** Blast animals spent significantly more time within the enclosed arm than the open arm of the EPM 2 weeks following injury. There was no significant preference for the central zone of the arena. **(C)** Similarly, 52 weeks following repeated blast exposure, data shows that blast animals spent more time within the enclosed arm than the open arm of the EPM. No significant preference for the central zone was observed for either blast or sham animals. *p < 0.05,***p < 0.001,****p < 0.0001, Data is represented as Mean ± SEM. EPM, Elevated Plus Maze.

At 2- and 52-weeks following blast injury, animals underwent the EPM assessment. While the animals freely explored the maze, their behavior was recorded using a video camera and tracked using the Ethovision software (Figure 2A). Two weeks following injury, blast animals spent significantly less time in the open arms of the maze in comparison to their sham counterparts, favoring the enclosed arms of the maze (*p* < 0.05; Figure 2B). Neither blast nor sham animals showed a preference for the center (the intersecting portion of the maze). Fifty-two weeks following injury, the test was repeated and blast animals again spent significantly less time in the open arm portion of the maze than sham animals, favoring the enclosed arms of the maze (Figure 2C).

### Animals Exposed to Repeated Blast Exposure Show Reduced Sociability

**Figure 3:**
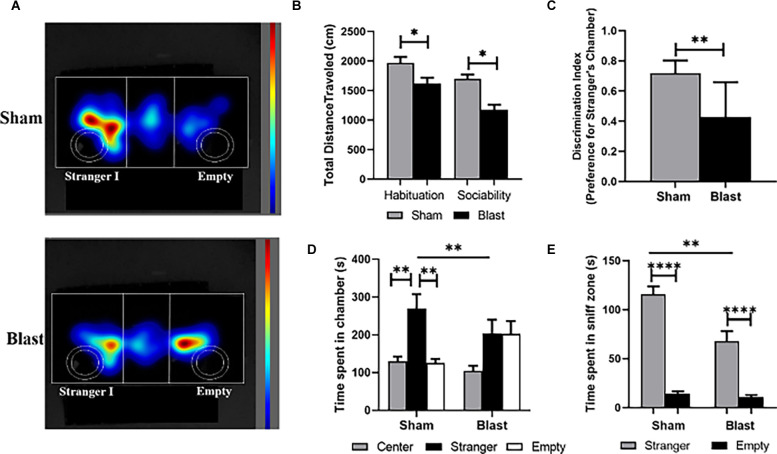
Animals exhibit decreases in sociability 36 following repeated blast exposure. **(A)** Representative heat map images show that sham animals spent more time with stranger I, whereas blast animals spent an equal amount of time in the chamber of stranger I and the empty chamber. The circular outlines indicate the sniff zones within each chamber. **(B)** Total distance traveled was significantly decreased for both the habituation and sociability trial of blast animals compared to shams. **(C)** An average discrimination index showed that sham animals had a significant preference for the stranger’s chamber (ratio > 0.5) than blast animals (ratio < 0.5). **(D)** Decreased sociability in blast animals was also indicated as no significant differences being observed in time spent in the chamber of stranger I and the empty chamber. **(E)** While both sham and blast animals spent significantly more time in the sniff zone of stranger I than the empty sniff zone, this time was still significantly decreased in blast animals compared to shams. *p < 0.05, **p < 0.01, ****p < 0.0001. Data is represented Mean ± SEM.

At 36 weeks following blast injury the three-chamber sociability test was used to quantify deficits in social behavior (Figure 3A). No obvious signs of aggression (i.e., biting or fighting) were observed in any of the rats during the task. The total distance traveled during both the habituation and sociability trials was significantly lower in comparison to sham animals (Figure 3B). A discrimination index showed that the preference for the stranger’s chamber was significantly higher for the sham group compared to blast (*p* < 0.01; Figure 3C). A one-way ANOVA analysis indicated that sham animals spent significantly more time in the chamber with the stranger than they did in the center or empty chamber (*F*_(2, 27)_ = 56.26; *p* < 0.01; Figure 3D). In comparison, the time blast animals spent within the empty chamber and the chamber with stranger I was equal, and there were no significant differences in the amount of time spent in any chamber (*F*_(2, 29)_ = 3.406) The time that sham animals spent in the chamber of the stranger was also significantly higher than for blast animals. Furthermore, a student’s t-test indicated that both blast and sham animals spent more time in the sniff zone of the stranger rat vs. the empty sniff zone (*p* < 0.0001), but sham animals spent significantly more time within the stranger’s sniff zone than blast animals (*p* < 0.01; Figure 3E).

### Repeated Blast Exposure Leads to Neuronal Dysfunction in the MC and Hippocampus

**Figure 4:**
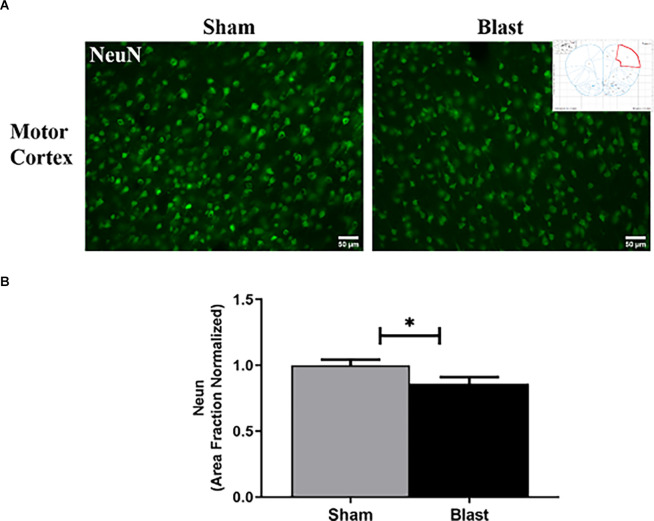
Blast restrains protein expression of NeuN in the MC 52 weeks following repeated blast exposure. **(A)** Representative images of NeuN in the MC region of the brain. Magnification is at 20x and scale bar = 50 μm. **(B)** Decreases in area fraction were observed in the MC of blast animals compared to shams. *p < 0.05. Data is represented as Mean ± SEM. MC, motor cortex.

**Figure 5:**
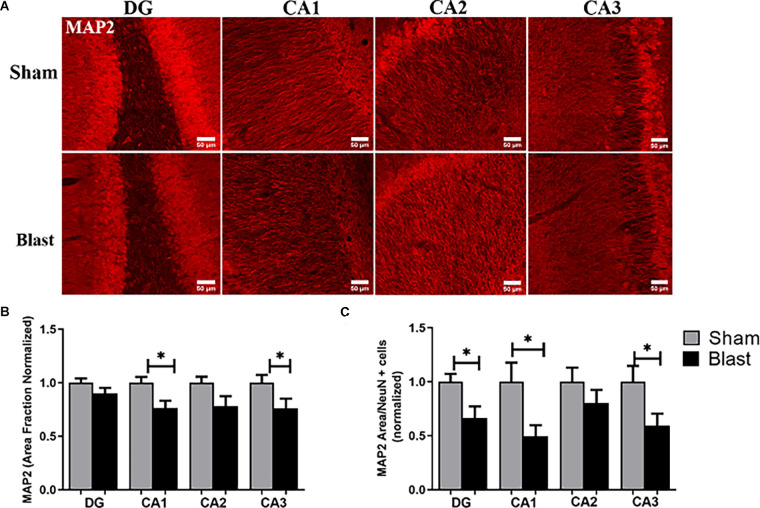
Dendritic alterations depicted dysfunction in the hippocampus 52 weeks following repeated blast exposure. **(A)** Representative images of MAP2 in the hippocampus region of the brain. Magnification is at 20x and scale bar = 50 μm. **(B)** Decreases in area fraction of MAP2 were observed in the CA1 and CA3 sub-regions of the hippocampus in blast animals compared to the sham group. **(C)** The MAP2 area divided by the amount of NeuN+ cells within the region of interest was significantly lower in the DG, CA1, and CA3 sub-regions of the hippocampus in the blast group. *p < 0.05. Data is represented as Mean ± SEM. MAP2, Microtubule-AssociatedProteins.

Microtubule-associated protein (MAP2) is a dendritic marker for neurons while NeuN stains the nuclei of mature neurons. Both markers aid in depicting patterns of neuronal loss and dysfunction in specific regions of the brain following injury. MAP2 and NeuN were measured by area fraction, which quantifies the amount of positive signal in the region of interest (Figure 4A). To assess the combination of neuronal and dendritic loss/dysfunction, the amount of MAP2 divided by the amount of positive NeuN cells in the region of interest was quantified. In the MC, the area fraction of NeuN was significantly decreased in blast animals with a 14% reduction compared to shams (*p* < 0.05; Figure 4B). In the hippocampus area fraction of MAP2 was significantly lower in the CA1 and CA3 regions of the hippocampus in blast animals with a 23% reduction compared to the sham group (Figures 5A,B). The MAP2 area divided by the number of NeuN+ cells was significantly lower in the DG, CA1, and CA3 regions of the hippocampus in blast animals in comparison to shams (Figure 5C).

### Decreased Levels of GFAP Were Observed Within the MC and Hippocampus Regions of Blast Animals

**Figure 6:**
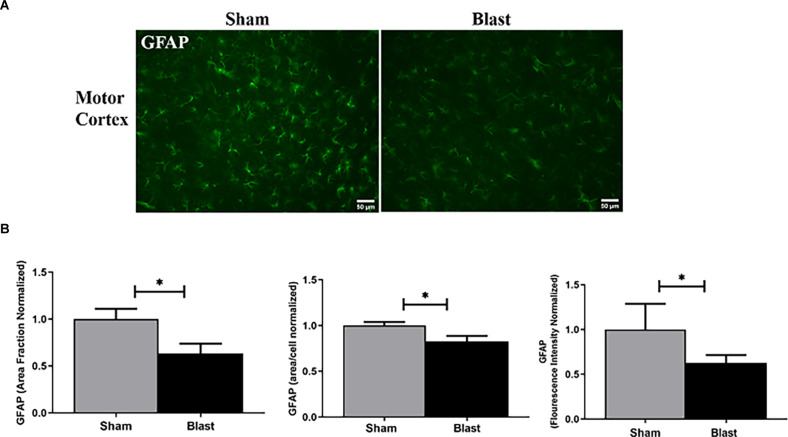
Astrocyte pathology was observed in the MC 52 weeks following blast injury. **(A)** Representative images of GFAP obtained from the MC region of the brain. Magnification is at 20x and scale bar = 50 μm. **(B)** Significant decreases in area fraction, mean area per cell, and fluorescence intensity in the MC were observed in blast animals compared to the sham group. *p < 0.05. Data is represented as Mean ± SEM. GFAP, glial-fibrillary acid protein.

One way that astrocyte reactivity was observed was through the measurement of GFAP expression within the brain (Figure 6A). The area fraction, which quantifies the amount of GFAP positive signal in the region of interest was measured. GFAP area fraction was significantly decreased (*p* < 0.05) in the MC of blast animals with a 37% reduction in comparison to shams. The mean area per cell and fluorescence intensity was also significantly decreased in blast animals compared to shams (Figure 6B).

**Figure 7:**
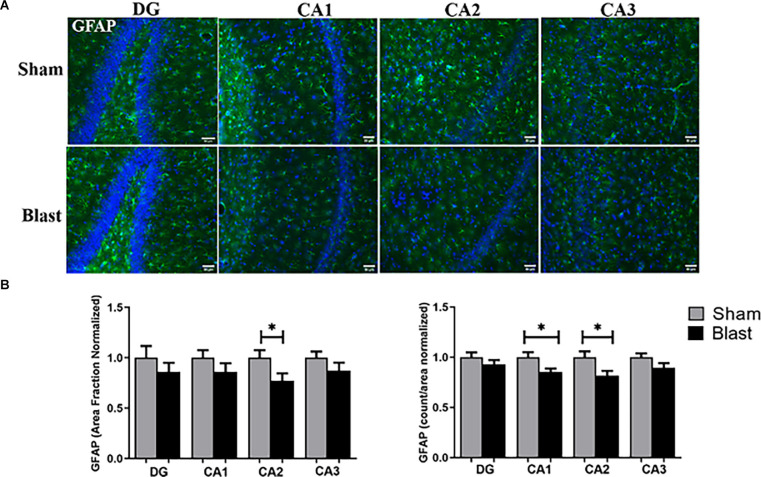
Decreased levels of GFAP were observed in the hippocampus 52 weeks following blast injury. **(A)** Representative images of GFAP (green) and DAPI (blue) obtained from the hippocampus region of the brain. Magnification is at 20x and scale bar = 50 μm. **(B)** Decreased positive GFAP signal (area fraction) was observed in the CA2 sub-region of the hippocampus in blast animals compared to shams. Decreases in the amount of GFAP+ astrocytes were observed in the CA1 and CA2 sub-regions in blast animals. *p < 0.05. Data is represented as Mean ± SEM. DAPI, 6-diamidino-2-phenylindole.

Within the hippocampus, similar downward trends were observed in the sub-regions (Figure 7A). For example, the area fraction showed a significant decrease in the levels of GFAP within the CA2 region of the hippocampus with a 23% reduction in comparison to shams. Quantification of the actual number of cells in the hippocampus (count per area), showed significantly fewer GFAP+ astrocytes within the CA1 and CA2 regions of the hippocampus in blast animals compared to shams (Figure 7B).

### Astrocyte Integrity Is Compromised Following Repeated Blast Exposure

**Figure 8:**
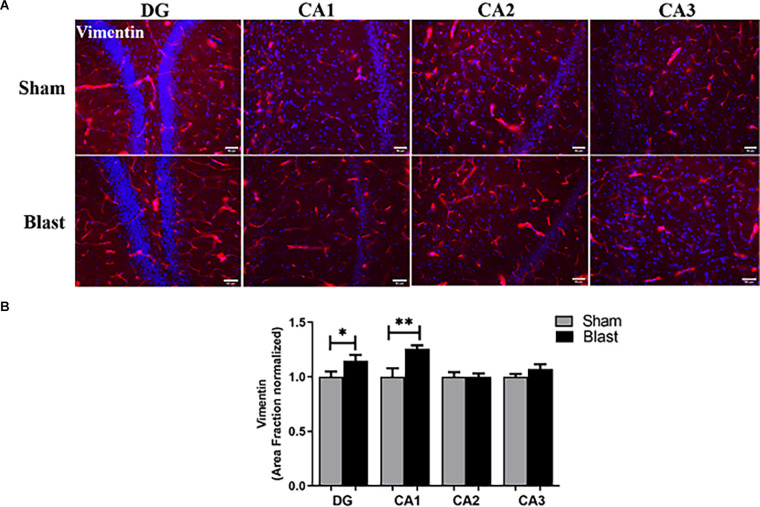
Gliosis was observed in the hippocampus 52 weeks following blast injury. **(A)** Representative images of Vimentin (red) and DAPI (blue) obtained from the hippocampus. Magnification is at 20x and scale bar = 50 μm. **(B)** Area fraction of vimentin was significantly increased in the DG and CA1 regions of the hippocampus in blast animals compared to shams. *p < 0.05, **p < 0.01. Data is represented as Mean ± SEM.

Astrogliosis was assessed by the changes in vimentin expression, which is an interfilament protein responsible for supporting cell integrity and is expressed in reactive astrocytes (Lopez-Rodriguez et al., 2015; Figure 8A). While levels of GFAP+ astrocytes seemed to have decreased in the hippocampus, increased levels of vimentin were observed in this region of the brain. Specifically, the area fraction was significantly increased in the DG and CA1 regions of the hippocampus in the blast group (Figure 8B). No significance in area fraction of vimentin was found in the MC between the blast and the sham groups.

### Astrocyte and Neuronal Pathology Were Correlated With Anxiety-Like Behaviors Following Repeated Blast Exposure

**Figure 9:**
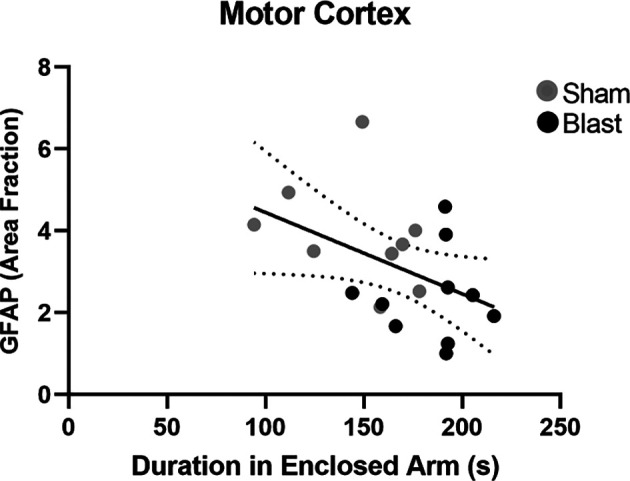
There is an association between chronic astrocyte pathology and anxiety-like behaviors in the motor cortex 52 weeks following injury. Analysis of correlation indicated a significant association between the area fraction of GFAP in the MC and the time spent in the enclosed arm of the EPM arena 52 weeks following repeated blast exposure (*p* = 0.05, *r* = −0.5). Overall regression lines are solid with 95% confidence intervals denoted with the black dotted lines.

**Figure 10:**
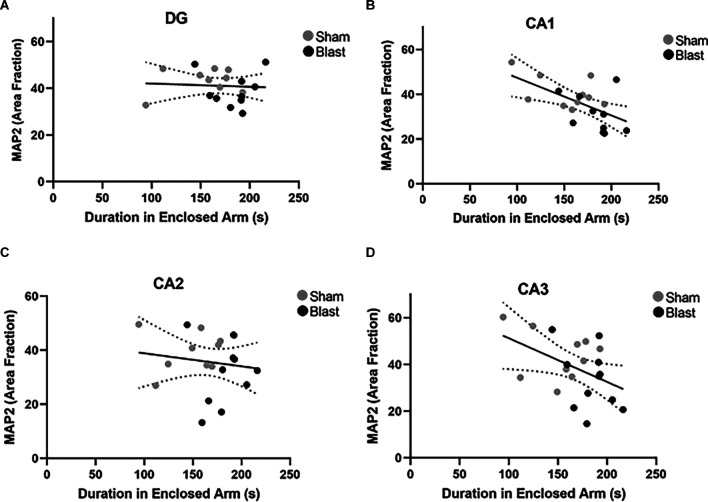
Dendritic alterations correlate with anxiety-like behaviors 52 weeks following blast exposure. Pearson’s correlation analysis between area fraction of MAP2 in the hippocampus and time spent in the enclosed arm of the EPM arena **(A–D)**. **(A)** No significant correlation was found between area fraction of MAP2 in the DG and time spent in the enclosed arm (*p* = 0.79, *r* = −0.06). **(B)** A significant negative correlation between area fraction of MAP2 in the CA1 and duration in the enclosed arm (*p* = 0.007, *r* = −0.6). **(C)** No significant correlation was found between area fraction of MAP2 in the CA2 and duration in the enclosed arm (*p* = 0.55, *r* = −0.14). **(D)** Correlation between area fraction of MAP2 in the CA3 and time spent in the enclosed arm was found to be significant (*p* = 0.04 *r* = −0.5). Overall regression lines are black with 95% confidence intervals denoted with black dotted lines.

Potential correlations between MAP2 and GFAP positive signal (area fraction) and anxiety-like behaviors (time spent in the enclosed, safe arm of the EPM) were investigated. A significant negative correlation between area fraction of GFAP in the MC and time spent in the enclosed arm of the EPM 52 weeks following injury was observed (*p* = 0.05, *r* = −0.5; Figure 9). Pearson’s correlation analysis also indicated significant negative correlations in the hippocampus (Figures 10A–D). Specifically, a negative correlation between area fraction of MAP2 and time spent in the enclosed arm was found to be significant in the CA1 (*p* = 0.007, *r* = −0.6; Figure 10B) and the CA3 (*p* = 0.04, *r* = −0.5; Figure 10D) sub-regions of the hippocampus. No significant correlations were found between MAP2 area fraction within the DG and CA2 sub-regions and time spent within the enclosed arm of the EPM.

### Decreased GFAP Expression in the Hippocampus Was Correlated With Sociability Deficits Following Blast Exposure

**Figure 11:**
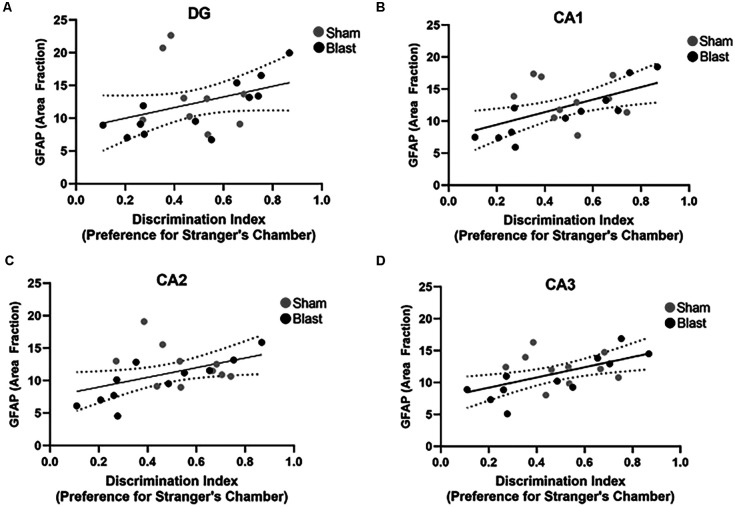
Decreased GFAP expression in the hippocampus correlates with sociability deficits 52 weeks following blast injury. Pearson’s correlation analysis between the area fraction of GFAP in the hippocampus and preference for the stranger’s chamber using the three-chamber sociability test **(A–D)**. **(A)** No significant correlation was found between the area fraction of GFAP in the DG and preference for the stranger’s chamber (*p* = 0.09, *r* = 0.4). **(B)** A significant positive correlation between the area fraction of GFAP in the CA1 and the preference for the stranger’s chamber (*p* = 0.009, *r* = 0.5). **(C)** A significant positive correlation was also found between the area fraction of GFAP in the CA2 and preference for the stranger’s chamber (*p* = 0.03, *r* = 0.5). **(D)** Correlation between the area fraction of GFAP in the CA3 and preference for the stranger’s chamber was also found to be significant (*p* = 0.009, *r* = 0.5). Overall regression lines are black with 95% confidence intervals denoted with black dotted lines.

Correlations between area fraction of GFAP and preference for the stranger’s chamber over the empty chamber during the three-chamber sociability test were examined (Figures 11A–D). Significant positive correlations were found within the CA1 (*p* = 0.009, *r* = 0.5; Figure 11B), the CA2 (*p* = 0.03, *r* = 0.5; Figure 11C), and the CA3 (*p* = 0.009, *r* = 0.5; Figure 11D) sub-regions of the hippocampus. A significant correlation was not observed between the GFAP area fraction in the DG and the preference for the stranger’s chamber.

## Discussion

With neurologic and psychiatric impairments being diagnosed following bTBI, it is important to fundamentally study these consequences that affect the Veteran community. In this study, animals were subjected to repetitive blasts and were found to display anxiety- and depression-like behaviors at 2-, 36-, and 52-weeks following blast injury. These behaviors were associated with astrogliosis and neuronal dysfunction within the hippocampus and MC. Results from this preclinical study indicate that blast exposures lead to long-term consequences like the behavioral outcomes reported within combat military personnel and Veterans.

In the current study, EPM was performed 2- and 52-weeks following repeated blast exposure with anxiety-like behaviors being observed. Typically, animals expressing more anxiety-like behaviors spend more time in the “safer” enclosed arms than in the more “dangerous” open arms, which was indicated in blast animals both 2- and 52-weeks following injury. Increased anxiety-like behavior has been shown in multiple rodent models of bTBI, with most evaluating at acute or subacute (<4 weeks) time points. In particular, a study by Sweis et al. (2016) used EPM to analyze anxiety-like behaviors 3, 6-, 24-, 72-, and 168-h following blast injury. Animals were subjected to five shock wave pulses focused on the frontal lobe area of the brain. Their results indicated significant differences in the EPM anxiety score index longitudinally between the blast groups, as well as between blast and shams. While their blast protocol differed from the current study, anxiety-like behaviors were seen in the cortex of blast animals at acute time points. Focusing on chronic behavioral outcomes in our current study indicated that these behaviors can persist long-term, and investigation of the MC adds further data to the pathological contribution to these deficits.

Acute and subacute studies show that anxiety is an outcome following bTBI (Sajja et al., 2015a, b; Hubbard et al., 2018). Chronic longitudinal (<24 weeks post-blast) assessments for anxiety and other behavioral outcomes such as depression are lacking. A study by Blaze et al. (2020) focused on linking long-term anxiety-related phenotypes in the amygdala with repeated bTBI. Their investigation included characterizing transcriptome-wide gene expression in the amygdala, then correlating it with fear and anxiety seen at both subacute (~4 weeks) and chronic (~52 weeks) time points. They found anxiety-like behaviors at the subacute time point (*via* elevated zero maze and open field assays), however, these behaviors diminished by 52 weeks following repeated blast exposure. Brains examined at both time points showed an interaction between injury and time points for genes that are implicated in neurodegeneration. The behavioral results differed from those of our studies, as we found anxiety-like behaviors 52 weeks following repeated blast exposure through EPM. Variations in their blast protocol (three blast exposures separated by 24 h and lower pressures [~75 kPa (10.5 psi)], behavioral assessments, and pathological assessments likely contribute to the differing results. Thus, it is necessary to dive deeper into how variation in blast protocols contributes to the complex behavioral and neuropathological outcomes.

The three-chamber sociability test assesses anxiety and depression in the form of general sociability in rodent models of CNS disorders (Nadler et al., 2004; Kaidanovich-Beilin et al., 2011; Johnson et al., 2013; Lopatina et al., 2014; Rein et al., 2020; Becker et al., 2021). Because rodents normally prefer to spend more time with another rodent (sociability), the three-chamber test can help identify rodents with deficits in sociability. The lack of social interactions is an accepted marker of various neurodevelopmental disorders as well as mental illnesses such as depression and schizophrenia, as well as anxiety, a behavioral deficit that can be a symptom of these illnesses (Moy et al., 2004, 2009; Lopatina et al., 2014; McKibben et al., 2014; Jaehne et al., 2015; Tough et al., 2017; Berger et al., 2019; Leclercq et al., 2020). Few TBI studies have used this tool (three-chamber sociability test) to evaluate deficits in social behavior, relating to depression. In a preclinical model of pediatric TBI, the three-chamber test was used to evaluate social outcomes following a controlled cortical impact. The study measured these social outcomes at adolescence (p35–42) and then again in early adulthood (p60–70; Semple et al., 2012). The premise for the use of this test was to prove that deficits in social interactions parallel the development of other behavioral deficits such as hyperactivity, a symptom of depression, and elevated anxiety. Results indicated decreased sociability in early adulthood whereas no significant differences in sociability were found in the adolescent animals. While the goal of their study was to show that age differences, as well as TBI, influenced social outcomes, this may be indicative that anxiety and depression develop with time or can be amplified chronically following brain injury. Collins et al. (2020) investigated sociability deficits following a single bTBI in mice. Animals underwent the three-chamber sociability test with decreases in sociability being observed 10 days following injury, but not present at 30 days following injury (Collins et al., 2020). Their rationale for this is that bTBI elicits time-dependent alterations in 5-HT architecture, a serotonin receptor that plays a role in depression, which coincides with altered social function observed in their data. This supports the need for more longitudinal long-term behavioral studies to understand whether pathological changes are time-dependent following blast injury. To our knowledge, these two TBI studies are the only research studies that have reported the social outcomes *via* the three-chamber sociability test, showing a growing need to understand how these outcomes may relate to chronic psychiatric impairments following blast injury.

In our study, results from the three-chamber sociability test indicated that blast animals were not able to perform the task like their sham counterparts. This was quantified through the significant decrease in total distance traveled in blast animals throughout all three chambers for the habituation and sociability trials. Additionally, during the sociability trials, blast animals spent less time with the unfamiliar rat than sham animals, which may be indicative of social anxiety, a common symptom of depression. While it has been documented that post-traumatic traits such as depression have been reported in the military population following blast injury, characterizing this in a preclinical blast model is limited.

The pathophysiology of the amygdala has been a focal point of research investigations and is shown to play a significant role in anxiety-like behaviors following bTBI, with limited knowledge on what regions of the brain play a role in depression. Clinical studies have found that the hippocampal-cingulate network pathology has a role in psychiatric impairments of anxiety and depression, with the cingulate gyrus having extensive connections to the MC (MacQueen and Frodl, 2011; Guo et al., 2015). As the hippocampus and MC play a vital role in memory encoding, explicit processing, and cognition; abnormalities in these stress-sensitive regions may be a cause of social anxiety and depression (Campbell and MacQueen, 2004). A clinical study by Roddy et al. showed that patients with MDD had low hippocampal gray matter, with the CA1 highlighted in the progression of the disease (Roddy et al., 2019). Decreased dendritic spines in the hippocampus have also been associated with mood and anxiety disorders. A study by Ma et al. (2021) observed decreased dendritic spine density and synaptic dysfunction within the hippocampus and suggested that this contributed heavily to depressive-like behaviors. Decreased neuron excitability in the MC has also been associated with patients with major depression (Cantone et al., 2017). A study by Peng et al. (2015) reported, through MRI measurements, that deficits in regions associated with cognition and motor processing (supplementary motor and premotor cortex) were observed in patients with MDD, especially in late-onset depression. Further, the cortex showed decreasing cortical thickness. Deep brain stimulation studies have linked long-term cognitive dysfunction associated with the motor and prefrontal cortices with long-term psychological impairments such as depression in TBI patients (Rahimpour and Lad, 2016). Further, studies by Wassermann et al. (2001) and Pallanti et al. (2010) have shown that inhibition of neural circuits in the MC played a leading role in patients with social anxiety disorder, as well as other anxiety-related personality traits.

As the current study demonstrated that decreases in MAP2, a protein expressed in dendrites, was observed within the CA1 and CA3 sub-regions of the hippocampus, this aligns with previous psychiatric studies showing the significant role the pathophysiology of the hippocampus plays in depression. In addition, an increase in the expression of vimentin was found. Vimentin expression in astrocytes following spinal cord injury and TBI has been proposed to act as a neurotrophic factor, as it enhances axonal growth, dendritic remodeling, as well as increased astrocyte integrity (Ramos et al., 2020; Stankevicins et al., 2020). This evidence supports that reactive astrocytes increase their expression of vimentin to play a neuroprotective role following blast injury. A study by Cobb et al. (2016) observed decreases in GFAP+ astrocytes in the left hippocampi in MDD, suggesting that GFAP+ astrocyte contributions to neuronal function may be compromised in depressed subjects, exacerbating the disease. As GFAP+ astrocytes were decreased within the hippocampus of blast animals in our study, this could indicate a link between hippocampal pathology and depressive-like behaviors following blast exposure. Our study also observed both decreases in levels of NeuN and GFAP within the MC at 52 weeks following repeated blast exposure. Decreases in the area of neuronal cells and GFAP expression suggest that cell death or other gray matter changes such as decreased cortical thickness are taking place, enhancing depressive behavioral outcomes. Furthermore, as astrocytes decrease and/or become dysfunctional, this may disrupt the tripartite synaptic structure between astrocytes and neurons. Because of this, communication between neurons, such as neurotransmitter release, could decrease in the MC, contributing to anxiety-like and depressive-like behaviors.

To establish a relationship between pathology and behavior, we investigated whether astrocyte reactivity and neuronal dysfunction were correlated with anxiety- and/or depression like behaviors. We found that decreased expression of GFAP and MAP2 in the MC and hippocampus negatively correlated with time spent within the enclosed arm of the EPM. This indicated that animals with lower levels of GFAP and MAP2 exhibited greater anxiety-like behaviors. Furthermore, increased levels of GFAP in the hippocampus were positively correlated with a preference for the stranger’s chamber over the empty chamber (ratio > 0.5), indicating that animals with lower expression of GFAP exhibited greater depression-like behaviors (ratio < 0.5). Collectively, the correlation analysis suggests that chronic alterations of astrocytes and dendrites within the hippocampus and MC are associated with anxiety-like and depression-like behaviors 52 weeks post-injury. This data is novel as these behaviors have been extensively linked to pathology within the amygdala, thus expanding our understanding of the regional connectivity within the brain. Additional research into axonal damage, dendrite morphology, and neurochemistry within these regions following blast are needed to further delineate the pathological mechanisms involved in the manifestation of anxiety and depression.

This chronic blast study is the first to investigate pathological changes in the hippocampus and MC and their potential link to long-term neuropsychiatric impairments. Due to the lack of research conducted on these two important brain regions, and their role in anxiety- and depression-like behaviors following blast exposure, understanding the mechanistic pathways within these regions, and how they may elicit chronic behavioral deficits following injury is imperative. Future directions include cell-specific gene expression studies within these two regions following blast injury and examining how their downstream effects play a role in anxiety and depression. Moreover, as evidence points to pathological changes in either the hippocampus and/or MC leading to psychiatric impairments following blast exposure, further studies are needed to understand whether these two regions work together or independently of each other in causing this behavioral demise. Additionally, expanding the behavioral assessment profile within chronic injury models to further characterize mood disorders will allow for a robust analysis of the psychiatric impairments that plague the Veteran community. Overall, this study provided crucial insight into the long-term neuropsychiatric impairments that have been reported by active military personnel and Veterans returning from combat. These findings will allow for the generation of applicable data that will aid in the development of therapeutic interventions, diagnostic tools, and other medical approaches that will lead to improved healthcare and quality of life for bTBI patients.

## Data Availability Statement

The raw data supporting the conclusions of this article will be made available by the authors, without undue reservation.

## Ethics Statement

The animal study was reviewed and approved by Virginia Tech Institutional Animal Care and Use Committee.

## Author Contributions

MD was responsible for analysis of results, interpretation of results, and preparation of manuscript. SM was responsible for data collection, analysis of data, interpretation of results, and preparation of manuscript. MU and ZW were responsible for data collection and analysis of data. PV was responsible for study design, securing funding, data collection, interpretation of results, and preparation of manuscript. All authors contributed to the article and approved the submitted version.

## Conflict of Interest

The authors declare that the research was conducted in the absence of any commercial or financial relationships that could be construed as a potential conflict of interest.

## Publisher’s Note

All claims expressed in this article are solely those of the authors and do not necessarily represent those of their affiliated organizations, or those of the publisher, the editors and the reviewers. Any product that may be evaluated in this article, or claim that may be made by its manufacturer, is not guaranteed or endorsed by the publisher.
